# Editorial: Gliomas microenvironment: new drug entities, mechanisms of action, molecular biomarkers and drug delivery strategies

**DOI:** 10.3389/fphar.2024.1431975

**Published:** 2024-06-05

**Authors:** Mariana Magalhães, Diana Matias, Claudia Carbone, Célia Cabral

**Affiliations:** ^1^ University of Coimbra, Institute for Interdisciplinary Research (IIIUC), Center for Neuroscience and Cell Biology (CNC), Coimbra, Portugal; ^2^ University of Coimbra, Coimbra Institute for Clinical and Biomedical Research (iCBR), Clinic Academic Center of Coimbra (CACC), Faculty of Medicine, Coimbra, Portugal; ^3^ University of Coimbra, Center for Innovative Biomedicine and Biotechnology (CIBB), Coimbra, Portugal; ^4^ Faculdade de Medicina, Instituto de Medicina Molecular João Lobo Antunes, Universidade de Lisboa, Lisbon, Portugal; ^5^ Department of Drug and Health Sciences, University of Catania, Catania, Italy; ^6^ University of Coimbra, Centre for Functional Ecology, Department of Life Sciences, Coimbra, Portugal

**Keywords:** gliomas, glioblastoma, tumor microenvironment, drug repurposing, drug delivery, biomarkers, nanotechnology

This Research Topic explores the tumor microenvironment of gliomas, highlighting the importance of understanding their molecular and cellular mechanisms for the development of personalized therapies with enhanced efficacy and tolerability. We have invited authors to contribute with original research and review articles, each providing insight into the experimental validation of novel bioactive molecules and the discovery of potential molecular biomarkers for tumor-specific therapies.

Among the submissions, we have selected 10 manuscripts of exceptional quality that include articles in the following categories: original research, review, systematic review, hypothesis and theory, perspective, and technology and code. These contributions cover a spectrum of human research methodologies, offering diverse perspectives on common themes.

Li et al. explored the molecular mechanisms underlying the potential efficacy of imipramine, an antidepressant, in the treatment of glioblastoma (GB). Using bioinformatics tools and human databases, the authors analyzed 77 samples from GB patients and 23 samples from healthy individuals. Their study identified the neuron-derived epidermal growth factor receptor (EGFR) as a potential target of imipramine, suggesting its role in modulating interactions between neurons, myeloid cells, and macrophages. Furthermore, they proposed the ability of imipramine to target EGFR mutants, potentially influencing GB treatment and drug resistance. These findings unveil novel avenues for targeted GB therapies.

Li and Xu conducted a comprehensive review on harnessing mitochondria as a therapeutic target in GB. The researchers explored natural bioactive compounds that target mitophagy and induce apoptosis via the mitochondrial pathway in GB models. Additionally, they discussed nanomedicine and sonodynamic therapy strategies to overcome biological barriers, including the blood-brain barrier (BBB), to improve drug delivery for targeted GB therapy. Their work highlights the potential of novel targeted therapies based on natural lead compounds to target mitochondria for improved GB treatment outcomes.

Wang and He conducted a comprehensive analysis of low-grade gliomas (LGGs), focusing on molecular subtypes, immune responses, and a prognostic signature linked to epithelial-mesenchymal transition (EMT) genes. Their study aimed to design new therapeutic strategies and discover new drugs for the treatment of LGGs. Employing a multi-omics approach and validating their findings *in vitro*, they revealed tumor heterogeneity driven by EMT-related gene expression variations. They categorized LGG-EMT into two subtypes with distinct clinical outcomes, clinicopathological features, mutational profiles, immune cell infiltration, and tumor microenvironment characteristics. This heterogeneity determines differential responses to immunotherapy and chemotherapy, guiding precise treatment decisions. Additionally, they developed a reliable EMT signature for LGG prognosis and identified potential small molecules to improve clinical outcomes. Their findings suggest a potential prognostic tool that is pivotal for personalized treatment strategies and prognostic assessment for LGGs.

Wang and He investigated the role of chloride intracellular channel 1 (CLIC1) in gliomas. Through a multi-omics approach and *in vitro* studies, they uncovered the therapeutic potential of targeting CLIC1 and elucidated its molecular mechanisms in low-grade gliomas (LGGs) and GB progression. Their findings suggest that elevated CLIC1 levels correlate with poorer survival outcomes in glioma patients. Knockdown experiments revealed CLIC1 as a potential treatment vulnerability and actionable target in gliomas, providing insights into prognostic factors and therapeutic avenues for glioma management.

Laws et al. have provided a fascinating insight into the potential mechanism by which primary cilia contribute to tumor-induced immunosuppression in GB. Their research, conducted with GB cell models and molecular techniques, revealed that suppressing CCRK, KIF3A, and IFT88 proteins led to the loss of primary cilia and decreased IL-6 levels. This study not only highlighted the involvement of primary cilia in IL-6 release and immunosuppression but also suggested a possible role in extracellular vesicle (EV) release. These findings could potentially revolutionize our understanding of GB and open new avenues for innovative therapeutic strategies targeting cilia.

Yang et al. delved into the connection between glioma and glycosylation. By employing bioinformatics methodologies alongside *in vitro* experiments, they elucidated the role of glycosylation modification in tumor progression, which is strongly correlated with glioma prognosis. The authors identified five prognosis-related genes implicated in glycosylation. Subsequently, they developed a predictive model using a glycosylation signature, which demonstrated high accuracy in predicting survival outcomes for glioma patients, validated through external datasets. Moreover, the glycosylation signature exhibited associations with immune infiltration in the glioma microenvironment, potentially indicating variable efficacy levels of immunotherapy. These insights promise significant contributions to future glioma research and clinical applications.

Zhang et al. investigated the prognostic significance of TGFB-induced factor homeobox 2 (TGIF2) in glioma, a study that holds potential implications for the fields of cancer biology and immunology. They examined TGIF2 expression in normal tissues and gliomas using public databases and analyzed its correlation with clinicopathologic features, prognosis, and potential biological functions in glioma patients, as well as its impact on tumor immune infiltration. *In vitro* studies further confirmed these findings. The results indicated that increased TGIF2 expression is strongly correlated with malignant phenotypes and a poor prognosis in glioma patients. Importantly, the knockdown of TGIF2 *in vitro* was shown to inhibit glioma invasion and migration and suppress the EMT phenotype. Thereby, these results suggest TGIF2 as a potential glioma biomarker linked to tumor immune infiltration and EMT, which may have direct implications for the development of novel therapeutic strategies for glioma.

Tripathy et al. conducted a review of the evolution of GB research, encompassing insights into GB biology and approved treatments over time. They explored the pivotal players within the GB microenvironment and the regulation of critical mechanisms involved in GB progression and survival. Furthermore, they examined the diverse array of potential therapeutic agents used for GB patients. This review underscores the imperative need for molecular targets and mechanisms that are specific to GB, particularly in the context of advancing personalized medicine, which holds immense potential for the future of GB treatment.

Li et al. developed a novel integrative stacking ensemble model framework (ecGBMsub), aimed at improving the classification of molecular subtypes in *IDH*-wild-type GB. Leveraging machine learning techniques, the researchers constructed an ensemble model using extrachromosomal circular DNA (eccDNA) molecular profiling. Additionally, they developed a user-friendly web tool that provides clinicians with a robust resource to discern molecular subtypes and explore potential links between GB subtypes and eccDNA biology. This advancement holds promise for helping clinicians predict molecular subtypes in GB patients, thereby facilitating more personalized treatment selection.

Jajin et al. conducted a systematic review and meta-analysis to investigate the effectiveness of vaccines in the treatment of GB. Their study examined the efficacy and safety of existing vaccines, including personalized vaccines, and compared them with conventional treatments for GB patients. The findings revealed that personalized vaccines exhibited superior efficacy in the treatment of GB, leading to improved overall survival, progression-free survival, and time to survival. Notably, AFTV, peptide, and dendritic cell-based vaccines emerged as effective options for glioma.

Taken together, these promising studies lay the groundwork for a better understanding of the molecular networks of GB, potentially refining the selection of personalized therapies for GB patients. This progress holds great promise for improving patient survival and overall wellbeing.

